# PET-FDG: Impetus

**DOI:** 10.3390/cancers12041030

**Published:** 2020-04-22

**Authors:** Cristina Nanni

**Affiliations:** Nuclear Medicine Bld.30, AOU S. Orsola-Malpighi, Via Massarenti n.9, 40138 Bologna, Italy; cristina.nanni@aosp.bo.it; Tel.: +39-051-214-3182; Fax: +39-051-636-3956

**Keywords:** multiple myeloma, FDG, PET, IMPeTUs, interpretation criteria

## Abstract

The International Myeloma Working Group (IMWG)recommends FDG PET/CT (Fluoro-Deoxy-glucose Positron Emission Tomography/Computed Tomography) as the gold standard imaging modality for initial evaluation and response to therapy assessment in multiple myeloma. In fact, FDG PET/CT, provides multiple useful indexes to risk-stratify patients and has significant prognostic value. However, multiple myeloma remains a complex disease to interpret on imaging. The Italian myeloma criteria for PET use (IMPeTUs) were proposed to standardize FDG PET/CT reading in multiple myeloma. In this communication an overview on IMPeTUs is provided as well as some examples of application.

## 1. Introduction

Multiple myeloma (MM)is an extremely complex oncological disease. The bone marrow (BM) is generally diffusely infiltrated by malignant plasma cell clones. Focal bone lesions and extramedullary (EM)involvement, with a possible spread all over the body, can be associated to bone marrow infiltrations. Epi-phenomena of the disease such as osteoporosis, bone fractures, spinal cord compression, anaemia, infections, and renal impairment can significantly impact outcome and imaging interpretation [1].

Nowadays magnetic resonance imaging (MRI) is considered the imaging gold-standard method for the detection of bone marrow involvement in MM. One of the major advantages of MRI is the ability to discriminate between myelomatous and normal bone marrow, allowing to differentiate myeloma from osteoporotic fractures in more than 90% of cases. In addition, MRI has the ability to localize spinal cord and nerve root compression, as well as the presence of soft tissue extension and/or extramedullary plasmacytomas. Finally, MRI is the procedure of choice in order to confirm a diagnosis of solitary plasmacytoma, by excluding the presence of additional disease, and can also assess tumor burden in patients diagnosed with nonsecretory or oligosecretory myeloma.

Magnetic resonance imaging (MRI) allows to directly visualize bone marrow infiltration much earlier than myeloma-related bone destruction and it is considered the imaging gold-standard method for the detection of myeloma bone marrow involvement. MRI is the procedure of choice to evaluate painful lesions and discriminate between benign and malignant vertebral fractures; it also has the ability to localize spinal cord and/or nerve root compression for surgical intervention or radiation therapy. Moreover, whole-body MRI is currently included in the diagnostic work-up of otherwise considered asymptomatic myeloma and solitary bone plasmacytoma, with remarkable therapeutic implications. However, despite its added value in the initial disease assessment, conventional MRI is of limited value in the evaluation of response after treatment since it only provides morphological information.

MM can present with variable metabolic uptake, ranging from low to extremely high, making interpretation more challenging especially at initial diagnosis [2,3].

There are in fact multiple elements that can contribute to misinterpretation of images.

Firstly, it is known that MM-related anaemia may cause a significant and diffuse increase in BM tracer uptake, by itself or in consequence to the administration of erythropoietin. This can determine a hot background in the bone, particularly in the spine, reducing small lesion detectability. Moreover, it can easily be confused with a similarly hypermetabolic diffuse bone marrow infiltration.

Secondly, FDG PET (Fluoro-Deoxy-glucose Positron Emission Tomography)-positive MM lesions may lay on morphologically normal bone on the corresponding CT (Computed Tomography) images and consequently may be classified as equivocal findings (especially if small), due to a reduced reader interpretation confidence.

Thirdly, the tendency of MM to produce bone focal lysis frequently causes fractures; reparative inflammation may appear falsely positive and can be confused with an active MM lesion [4,5]. 

Finally, bone metallic implants used to support the backbone and therefore reduce the risk of spinal fractures and spinal cord compression or to treat pathological fractures may cause significant artifacts on CT and PET images and may be a site of infection with resulting nonspecific FDG uptake. False positivity may also be associated to percutaneous vertebroplasty.

All these challenging aspects and pitfalls make interpretation challenging and require extensive experience and knowledge of the particular aspects of MM.

Different interpretation criteria have been proposed in literature over the past years. Some groups propose SUV (Standardized Uptake Value)-derived parameters such as total lesion glycolysis (TLG) and metabolic tumor volume (MTV) to assess the active disease burden at baseline and its variation (delta) as a consequence of therapy [6].A standard and widely accepted software program to harmonize MTV or TLG measure in clinical practice is however still lacking. Other researchers propose purely semi-quantitative or visual methods. All these interpretation criteria may provide different thresholds in terms of positivity in the case of unclear findings, but none of the proposed criteria has been clinically validated.

New visual descriptive criteria (Italian myeloma criteria for PET use or IMPeTUs) has been defined by a group of nuclear medicine experts, hematologists, and medical physicists for use in both clinical workflow and multicenter trials. IMPeTUs aim to standardize FDG PET/CT evaluation in MM patients thus harmonizing image interpretation [7]. It includes a visual interpretation that quantifies FDG uptake using the five-points scale of the Deauville score (DS),already in practice for interim and post-therapy FDG PET in lymphoma [8], in association with morphological and anatomical aspects of FDG distribution such as bone marrow non-focal uptake, focal bone lesions (site, number, and uptake), para-medullary, or extra-medullary lesions.

These criteria were validated as reproducible. Positivity cut-offs were recognized “a posteriori”, based on prospective Italian and French trials.

## 2. Impetus Criteria Description

IMPeTUs criteria are purely visual in order to minimize the impact of different technologies on scan interpretation. However, it is accepted to use lesion-to-background SUV ratio measurements to reinforce visual image interpretation.

The criteria are designed on images based on OSEM (Ordered Subset Expectation Maximization) reconstruction algorithms. No time-of-flight signal-to-noise ratio optimizing algorithms should be used while applying them. Variables include the description, using a five-point scale, of: metabolic state of the BM, number and site of focal PET positive lesions with or without osteolytic characteristics, presence and site of EM disease, presence of paramedullary (PM) disease, and presence of fractures. The visual degree of uptake is defined for the target lesion and EM lesions according to the schema proposed in the Deauville criteria for the evaluation of lymphoma patients [9].

In detail, the following features must be reported.

Diffuse bone marrow (BM) uptake:
Deauville criteria:
1 No uptake at all
2 ≤ mediastinal blood pool uptake
3 >mediastinal blood pool uptake, ≤ liver uptake
4 > liver uptake +10%
5 >> liver uptake (twice)
A^ appended if there is hypermetabolism in limbs and ribs [10]
Focal bone lesions (F):
Lesion number group (x):
x = 1: no lesions
x = 2: 1 to 3 lesions
x = 3: 4 to 10 lesions
x = 4: >10 lesions
S: skull
Sp: spine
Extra Sp: all the rest
Hottest focal bone lesion Deauville criteria (target lesion is the hottest area):
1 No uptake at all
2 ≤ mediastinal blood pool uptake
3 > mediastinal blood pool uptake, ≤ liver uptake
4 > liver uptake +10%
5 >> liver uptake (twice or more)
Lytic lesions(L) at CT associated to PET:
Lesion number group (x):
x = 1: no lesions
x = 2: 1 to 3 lesions
x = 3: 4 to 10 lesions
x = 4: >10 lesions
Presence of at least one fracture on CT images (Fr)
Presence of paramedullary disease (PM): a bone lesion involving surrounding soft tissues with bone cortical interruption
Extramedullary disease (EM):
Site
Nodal disease (N) plus site:
LC: laterocervical
SC: supraclavicular
M: mediastinal
Ax: axillary
Rp: retroperitoneal
Mes: mesentery
In: inguinal
Extranodal disease (EN) plus site:
Li: liver
Mus: muscle
Spl: spleen
Sk: skin
Oth: other
Plus, Deauville criteria for target EM lesions (target lesion is the hottest area):
1 No uptake at all
2 ≤ mediastinal blood pool uptake (SUV max)
3 > mediastinal blood pool uptake, ≤ liver uptake
4 > liver uptake + 10%
5 >> liver uptake (twice or more).

## 3. Concordance Analysis

Those criteria were promptly proved applicable in clinical practice. Despite MM being a complicated disease presenting with many different findings, IMPeTUs is easy enough to easily be reproduced. In total, 86 patients (211 FDG PET/CT scans) affected by symptomatic MM enrolled in the multi-center, phase III EMN (European Myeloma Network) 02 study and scanned with FDG PET/CT at baseline, after induction, and end of treatment were prospectively enrolled. The PET images were a posteriori reinterpreted by five expert readers according to a blinded independent central review (BICR) methodology. The percentage agreement was superior to 75% for all the time points, reaching 100% of agreement in assessing the presence of skull lesions after therapy. Comparable results were obtained when the agreement analysis was performed using the Krippendorff’s alpha coefficient either in every single time point of scanning or overall for all the scans together. DS proved highly reproducible; the highest reproducibility being demonstrated for score 4 [7].

## 4. Impetus Criteria: Examples of Application

**Figure 1:**
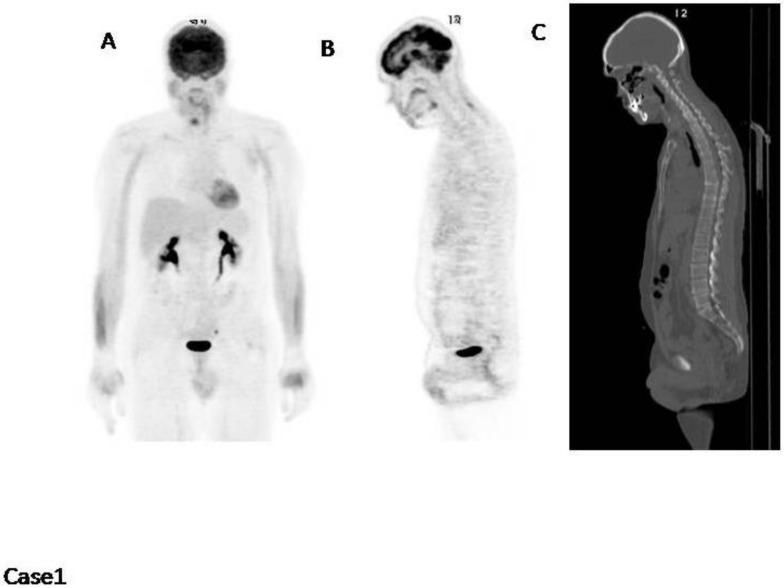
(**A**) MIP (Maximum Intensity Projection); (**B**) PET (Positron Emission Tomography) sagittal cut to highlight the bone marrow diffuse uptake; (**C**) CT (Computed Tomography) sagittal cut. No abnormal uptake is present.

Case1 (Figure 1): This patient is an example of a normal FDG PET/CT scan. No areas of increased uptake nor fractures or lytic lesions.

The only feature worthy of reporting is BM2 (bone marrow with Deauville score 2), which means that the bone marrow has a diffuse increased uptake within normal limits (uptake lower than the mediastinal pool).

**Figure 2:**
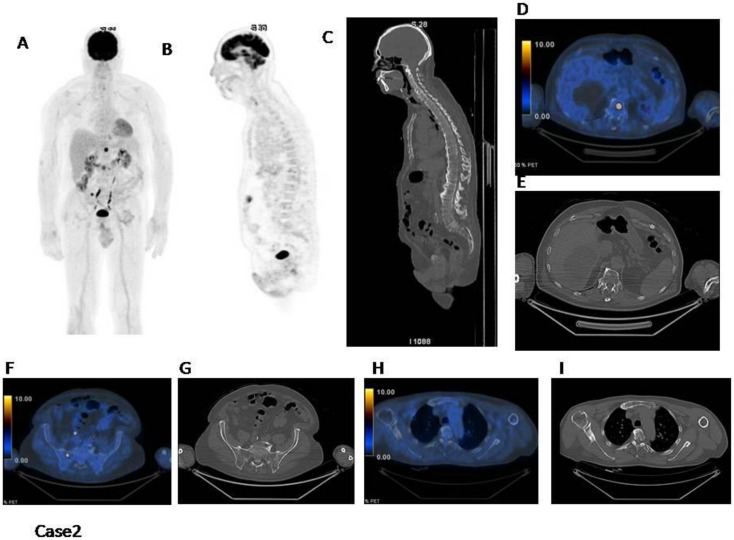
(**A**) MIP; (**B**) PET sagittal cut; (**C**) CT sagittal cut; (**D**–**I**) fused images and CT axial cut on different focal lesions. There are at least three focal hot spots with no underlying lytic lesions (D: vertebra, F: sacrum, H: right rib).

Case2 (Figure 2): This patient has a normal distribution in the bone marrow. However, there are at least three focal hot spots with no underlying lytic lesions. In this case IMPeTUs are: BM2 (normal bone marrow), F2 (three focal hot lesions) with DS4Sp (spinal) and ExP (extraspinal in the rib).

**Figure 3:**
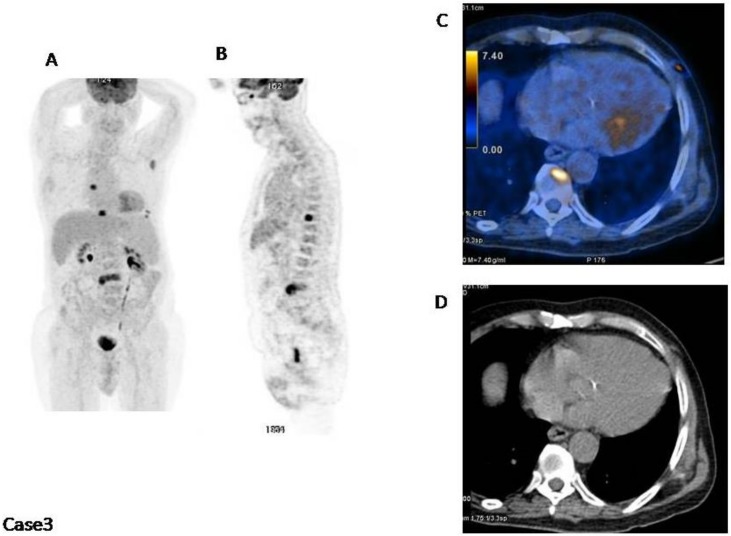
(**A**) MIP; (**B**) PET sagittal cut; (**C**) fused images axial cut; (**D**) CT axial cut. There are at least four focal hot spots with lytic lesions underlying and a subcutaneous lesion in the left thorax (C).

Case3 (Figure 3): This patient has a normal distribution in the bone marrow. However, there are at least four focal hot spots with lytic lesions underlying and a subcutaneous lesion in the left thorax. In this case IMPeTUs are:BM2 (normal bone marrow), F3 (four focal hot lesions) with DS4Sp (spinal) and ExP (extraspinal in the left scapula), L3 (four lytic lesions at CT), EM ENSk DS4 (one extramedullary extranodal lesion localized in the skin with DS4).

**Figure 4:**
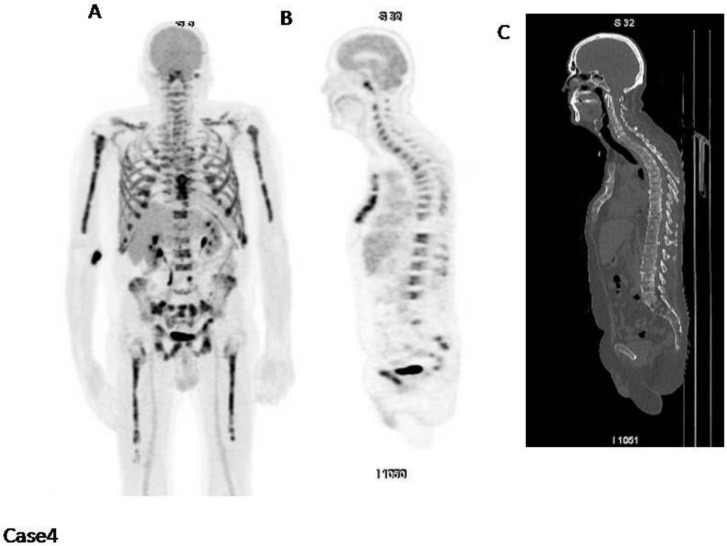
(**A**) MIP; (**B**) PET sagittal cut; (**C**) CT sagittal cut. There is a diffuse and severe bone marrow uptake in limbs, pelvis and spine.

Case4 (Figure 4): This patient has a diffused increased bone marrow uptake also involving limbs, with a fracture in T10. In this case IMPeTUs are: BM4 A (increased bone marrow uptake also in the limbs) and Fr (one fracture on CT).

**Figure 5:**
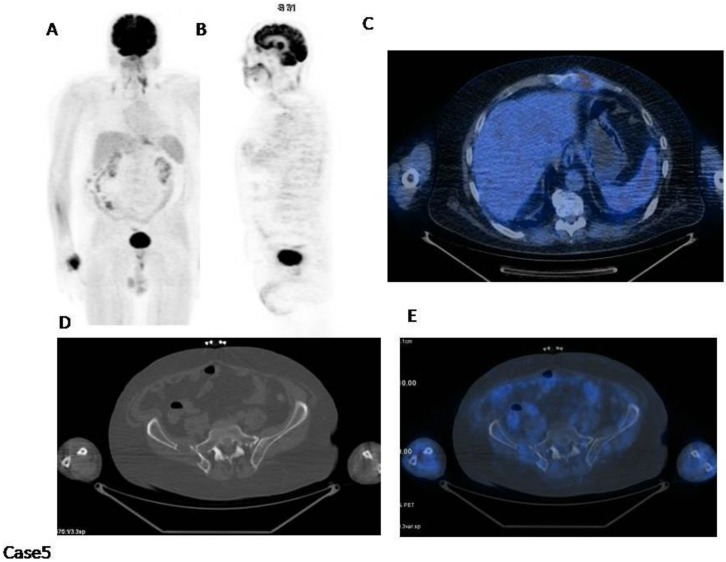
(**A**) MIP; (**B**) PET sagittal cut; (**C**) fused images axial cut; (**D**) CT axial cut; (**E**) fused images axial cut. There is a lytic lesion in the sternum involving surrounding tissues (C) and a cold lytic lesion in the right bony pelvis (E).

Case5 (Figure 5): This patient has a normal diffuse bone marrow uptake with a lytic lesion in the sternum involving surrounding tissues and a cold lytic lesion in the right bony pelvis.

In this case IMPeTUs are: BM2 (normal bone marrow), F2 (one focal hot lesion) with DS4 ExP (extraspinal in the sternum), L2 (two lytic lesions at CT), PM (at least one focal hot lesion involves the surrounding tissues).

## 5. Positivity Cut Offs

Other than being easily reproducible, impetus criteria were also used to define positivity cut-offs and therefore identify patients with active disease especially after therapy.

These positivity cut-offs were set a posteriori in a large and homogeneous patient population (228 pts) evaluated with FDG PET/CT before therapy and pre-maintenance in the light of follow up data.

This study concludes that patients who maintain a DS ≥ 4 at least in one focal lesion or DS ≥ 4 in the bone marrow has a significantly shorter progression-free survival and a significantly shorter overall survival [11].

## 6. Use of Impetus in Literature

In addition to the previously described positivity cut-offs, the prognostic role of those criteria has been further investigated in some studies.

For example, a comparison between the prognostic roles of different MM staging systems in 59 patients was performed. More specifically, the staging systems included the Durie–Salmon staging system (DSS), the Revised International Staging System (RISS) classification, and the Durie–Salmon plus (DS Plus) staging system based on IMPeTUs. The latter of these staging systems demonstrated significantly superior accuracy in stratifying patients at diagnosis.

Authors reported that on multivariate analysis DS Plus stage III (HR (Hazard Ratio): 11.539, *p* = 0.021) and the Deauville score of bone marrow ≥4 (HR: 3.487, *p* = 0.031) were independent prognostic factors associated with OS (Overall Survival). Both the DS Plus based on IMPeTUs and RISS possessed better potential in characterizing and stratifying MM patients compared with the DSS. Moreover, DS Plus stage III and the Deauville score of bone marrow ≥4 turned out to be reliable prognostic factors in newly diagnosed MM patients [12].

Another recently published study compared the prognostic value of IMPeTUs criteria to MTV and TLG calculated on the basis of a home-developed software on 47 patients with untreated MM. The authors found that MTV and TLG calculated on the basis of an in-house developed software for lesions with SUV max > 2.5 were both prognostic for PFS (Progression Free Survival) and OS while IMPeTUs and other well recognized prognostic parameters (such as the presence of extramedullary disease or SUV max) were not. This study partially confirms previous studies on IMPeTUs considering that their strongest prognostic value was found after therapy, and this time point was not included for comparison in the study [6].

## 7. Conclusions

IMPeTUs criteria were proposed to simplify and standardize multiple myeloma FDG PET/CT reporting. They are based on the Deauville score scale and were found to provide a significant prognostic index when the tracer uptake remains high in the bone marrow and/or hot focal lesions especially after therapy.
